# Supplementary material for: Il-6 signaling exacerbates hallmarks of chronic tendon disease by stimulating reparative fibroblasts
Source: eLife. 2025 Feb 7;12:RP87092. doi: 10.7554/eLife.87092 (PMC11805502; doi:10.7554/eLife.87092)
Supplement: Supplementary file 1. — GEO accession number, patient sex, source tissue, patient age, donor number, and disease state of the isolated tissue ordered by GEO accession number. Samples from sheathed tendons are strikethrough and were excluded from further analysis. [file elife-87092-supp1.docx]

| **GEO accession number** | **Sex** | **Source tissue** | **Age** | **Donor number** | **Disease state** |
| --- | --- | --- | --- | --- | --- |
| GSM639748 | Male | Non-sheathed tendon;Brachialis | 45 | 5 | Normal |
| ~~GSM639749~~ | ~~Male~~ | ~~Sheathed tendon;EDC~~ | ~~48~~ | ~~9~~ | ~~Normal~~ |
| GSM639750 | Male | Non-sheathed tendon;Quadriceps | 41 | 10 | Normal |
| ~~GSM639751~~ | ~~Male~~ | ~~Sheathed tendon;Flexor-Pronator~~ | ~~62~~ | ~~12~~ | ~~Normal~~ |
| GSM639752 | Female | Non-sheathed tendon;Subscapularis | 62 | 13 | Normal |
| GSM639753 | Male | Non-sheathed tendon;Patellar | 32 | 15 | Normal |
| GSM639754 | Female | Non-sheathed tendon;Subscapularis | 61 | 16 | Normal |
| GSM639755 | Male | Non-sheathed tendon;Subscapularis | 59 | 17 | Normal |
| ~~GSM639756~~ | ~~Male~~ | ~~Sheathed tendon;Flexor-Pronator~~ | ~~45~~ | ~~19~~ | ~~Normal~~ |
| GSM639757 | Female | Non-sheathed tendon;Subscapularis | 63 | 20 | Normal |
| GSM639758 | Female | Non-sheathed tendon;Biceps | 65 | 21 | Normal |
| GSM639759 | Female | Non-sheathed tendon;Subscapularis | 64 | 23 | Normal |
| GSM639760 | Male | Non-sheathed tendon;Biceps | 50 | 24 | Normal |
| ~~GSM639761~~ | ~~Male~~ | ~~Sheathed tendon;ECRB~~ | ~~55~~ | ~~26~~ | ~~Normal~~ |
| GSM639762 | Male | Non-sheathed tendon;Subscapularis | 50 | 27 | Normal |
| GSM639763 | Male | Non-sheathed tendon;Biceps | 41 | 28 | Normal |
| GSM639764 | Male | Non-sheathed tendon;Biceps | 46 | 29 | Normal |
| ~~GSM639765~~ | ~~Male~~ | ~~Sheathed tendon;ECRL~~ | ~~52~~ | ~~30~~ | ~~Normal~~ |
| GSM639766 | Male | Non-sheathed tendon;Subscapularis | 66 | 31 | Normal |
| GSM639767 | Female | Non-sheathed tendon;Teres minor | 46 | 32 | Normal |
| GSM639768 | Male | Non-sheathed tendon;Subscapularis | 44 | 33 | Normal |
| GSM639769 | Female | Non-sheathed tendon;Biceps | 59 | 34 | Normal |
| GSM639770 | Female | Non-sheathed tendon;Subscapularis | 49 | 35 | Normal |
| GSM639771 | Male | Non-sheathed tendon;Biceps | 45 | 5 | Tendinopathic |
| ~~GSM639772~~ | ~~Male~~ | ~~Sheathed tendon;ECRB~~ | ~~48~~ | ~~9~~ | ~~Tendinopathic~~ |
| GSM639773 | Male | Non-sheathed tendon;Patellar | 41 | 10 | Tendinopathic |
| ~~GSM639774~~ | ~~Male~~ | ~~Sheathed tendon;Flexor-Pronator~~ | ~~62~~ | ~~12~~ | ~~Tendinopathic~~ |
| GSM639775 | Female | Non-sheathed tendon;Suspraspinatus | 62 | 13 | Tendinopathic |
| GSM639776 | Male | Non-sheathed tendon;Patellar | 32 | 15 | Tendinopathic |
| GSM639777 | Female | Non-sheathed tendon;Suspraspinatus | 61 | 16 | Tendinopathic |
| GSM639778 | Male | Non-sheathed tendon;Suspraspinatus | 59 | 17 | Tendinopathic |
| ~~GSM639779~~ | ~~Male~~ | ~~Sheathed tendon;Flexor-Pronator~~ | ~~45~~ | ~~19~~ | ~~Tendinopathic~~ |
| GSM639780 | Female | Non-sheathed tendon;Suspraspinatus | 63 | 20 | Tendinopathic |
| GSM639781 | Female | Non-sheathed tendon;Suspraspinatus | 65 | 21 | Tendinopathic |
| GSM639782 | Female | Non-sheathed tendon;Suspraspinatus | 64 | 23 | Tendinopathic |
| GSM639783 | Male | Non-sheathed tendon;Suspraspinatus | 50 | 24 | Tendinopathic |
| ~~GSM639784~~ | ~~Male~~ | ~~Sheathed tendon;ECRB~~ | ~~55~~ | ~~26~~ | ~~Tendinopathic~~ |
| GSM639785 | Male | Non-sheathed tendon;Suspraspinatus | 50 | 27 | Tendinopathic |
| GSM639786 | Male | Non-sheathed tendon;Suspraspinatus | 41 | 28 | Tendinopathic |
| GSM639787 | Male | Non-sheathed tendon;Suspraspinatus | 46 | 29 | Tendinopathic |
| ~~GSM639788~~ | ~~Male~~ | ~~Sheathed tendon;ECRB~~ | ~~52~~ | ~~30~~ | ~~Tendinopathic~~ |
| GSM639789 | Male | Non-sheathed tendon;Suspraspinatus | 66 | 31 | Tendinopathic |
| GSM639790 | Female | Non-sheathed tendon;Suspraspinatus | 46 | 32 | Tendinopathic |
| GSM639791 | Male | Non-sheathed tendon;Suspraspinatus | 44 | 33 | Tendinopathic |
| GSM639792 | Female | Non-sheathed tendon;Suspraspinatus | 59 | 34 | Tendinopathic |
| GSM639793 | Female | Non-sheathed tendon;Suspraspinatus | 49 | 35 | Tendinopathic |
